# A Feasibility Study for Measuring Accurate Chest Compression Depth and Rate on Soft Surfaces Using Two Accelerometers and Spectral Analysis

**DOI:** 10.1155/2016/6596040

**Published:** 2016-11-24

**Authors:** Sofía Ruiz de Gauna, Digna M. González-Otero, Jesus Ruiz, J. J. Gutiérrez, James K. Russell

**Affiliations:** ^1^Department of Communications Engineering, University of the Basque Country (UPV/EHU), Alameda Urquijo S/N, 48013 Bilbao, Spain; ^2^Department of Emergency Medicine, Oregon Health & Science University, 3181 SW Sam Jackson Park Road, Portland, OR 97239, USA

## Abstract

*Background*. Cardiopulmonary resuscitation (CPR) feedback devices are being increasingly used. However, current accelerometer-based devices overestimate chest displacement when CPR is performed on soft surfaces, which may lead to insufficient compression depth.* Aim*. To assess the performance of a new algorithm for measuring compression depth and rate based on two accelerometers in a simulated resuscitation scenario.* Materials and Methods*. Compressions were provided to a manikin on two mattresses, foam and sprung, with and without a backboard. One accelerometer was placed on the chest and the second at the manikin's back. Chest displacement and mattress displacement were calculated from the spectral analysis of the corresponding acceleration every 2 seconds and subtracted to compute the actual sternal-spinal displacement. Compression rate was obtained from the chest acceleration.* Results*. Median unsigned error in depth was 2.1 mm (4.4%). Error was 2.4 mm in the foam and 1.7 mm in the sprung mattress (*p* < 0.001). Error was 3.1/2.0 mm and 1.8/1.6 mm with/without backboard for foam and sprung, respectively (*p* < 0.001). Median error in rate was 0.9 cpm (1.0%), with no significant differences between test conditions.* Conclusion*. The system provided accurate feedback on chest compression depth and rate on soft surfaces. Our solution compensated mattress displacement, avoiding overestimation of compression depth when CPR is performed on soft surfaces.

## 1. Introduction

Quality of cardiopulmonary resuscitation (CPR) is key to increase survival from cardiac arrest. Providing chest compressions with adequate rate and depth is difficult even for well-trained rescuers [1]. When cardiac arrest occurs in hospital, the patient is usually lying on a bed. Mattresses tend to deform and move downwards during CPR, thus reducing the efficiency of chest compressions [2]. The work required to perform chest compressions increases in proportion with the distance traveled by the rescuer's hands, so the compression of the mattress increases workload and consequently also rescuer fatigue [3].

Resuscitation guidelines recommend providing CPR on firm surfaces when possible [4, 5]. Transferring the patient to the floor would ensure a firm surface, but it cannot always be done safely and promptly. Another alternative would be the use of backboards, which can be placed beneath the patient during CPR to increase the area over which the compression force is spread and reduce the amount of mattress compression. However, it is not clear whether the use of backboards alone improves compression depth [6–9].

The deformation of the mattress during CPR is variable, dependent on factors such as target depth, patient weight, type of mattress, and the use of a backboard [3]. This makes it difficult for rescuers to assess whether they are providing chest compressions with an adequate depth. The use of monitoring and feedback devices during CPR can help rescuers to improve quality of chest compressions [10, 11]. However, devices that do not take into account the underlying mattress will overestimate compression depth [12]. Single accelerometer-based devices measure chest displacement. When chest compressions are provided on a mattress, they sense the sum of the chest compression (sternal-spinal displacement) plus the mattress deflection [12, 13]. Assuming that chest displacement corresponds to chest compression depth, these devices will incorrectly coach the rescuers, potentially causing too shallow chest compression. So far, accelerometer-based CPR feedback devices cannot perform accurately on soft surfaces.

In this study, we present a solution to provide feedback on compression depth and rate when compressions are delivered on soft surfaces. The system used two accelerometers: one was placed on the chest of the patient to measure chest displacement (sternal-spinal displacement plus mattress deflection) while the other was placed at the back of the patient to measure mattress deflection. To estimate compression depth and rate from acceleration, we applied an algorithm based on the spectral analysis of consecutive 2-second segments of the involved acceleration signals [14]. The system was evaluated in a simulated resuscitation scenario with different surfaces, CPR providers, and CPR conditions.

## 2. Materials and Methods

### 2.1. Study Design

The aim of the study was to quantify the error in the estimation of chest compression depth and rate during CPR performed on soft surfaces. For this assessment, we collected recordings using a* sensorized* manikin to provide the gold standard. Our secondary results were comparisons of the measured error as a function of several influencing factors: type of mattress, backboard use, and compression rates. To study the influence of the mattress, we used two models with different compositions, foam and sprung. We also wanted to study the influence of providing compressions with slower (80 cpm), recommended (100 cpm), and faster (120 cpm) rates, as this influences depth.

We designed our study as a randomized crossover study. Before starting the data collection, each participant practiced continuous chest compressions with the manikin placed on the mattress and their hands on the chest accelerometer. Then, we randomly grouped participants in couples and each couple performed 12 experiments: with each mattress with and without the backboard and for the three different compression rates. Each experiment consisted of 3-minute sessions with a first minute of continuous chest compressions, followed by a rescuer change, and a 2-minute series of 30 compression instances with 5-second pauses in between. Compressions were provided with the mattress placed on the floor and with rescuers kneeling beside the manikin. Target depth was always 50–60 mm and compression rate was guided using a metronome. The order of the experiments was randomized for each couple. Between consecutive experiments, rescuers had a 10-minute break. The ethical committee for research involving human subjects of the University of the Basque Country (CEISH UPV/EHU BOPV 32, 17-2-2014) approved the experimental protocol (M10-2015-208-RUIZ-OJEDA).

We calculated the sample size taking into account the standard deviation per record of the method reported in a previous study [15] and fixing a 95% confidence level and a margin of error lower than 3%. This yielded a sample size of 3 records (couples) per testing condition, but we fixed it to four for safety. The eight participants were selected randomly from a main group recruited for different ongoing studies on measuring CPR quality with accelerometers. They had no previous experience in CPR training. All of them attended a 2-hour CPR basics course including a period of training with the manikin placed on the floor. They were trained for a compression depth of 50–60 mm and a compression rate of 100 cpm (metronome guided). All of them signed the informed consent for the different experiments proposed, including this study on soft surfaces. The written informed consent was the only inclusion criterion.

### 2.2. Equipment and Data Collection

We used a CPR manikin torso (Resusci Anne CPR, Laerdal Medical AS, Stavanger, Norway) and placed a resistive sensor (SP1-4, Celesco Transducer Products Inc., Chatsworth, CA, USA) inside its chest to measure the reference chest displacement signal. We placed distributed weight plates inside the manikin increasing its weight up to 20 kg to provide a more realistic simulation of a human torso. For CPR experiments, we used two types of mattresses: foam (800 × 2000 × 90 mm, Pardo, Zaragoza, Spain) and sprung (900 × 1800 × 100 mm, Pardo, Zaragoza, Spain). Some experiments were conducted with a backboard (CPR Board, Ferno, Wilmington, OH, USA) placed between the mattress and the manikin (Figure 2).

**Figure 1:**
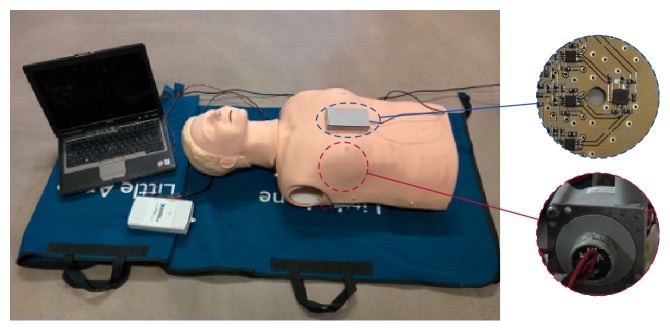
Experimental setup (I). The Resusci Anne manikin fitted with a resistive sensor (shown in the bottom circle). The two triaxial accelerometers encased in a metallic box: one is on the chest (shown in the top circle) and the other is on the floor. The acquisition card and the laptop computer are on the left.

**Figure 2:**
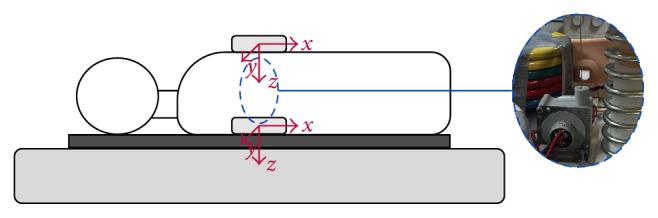
Experimental setup (II). The manikin was loaded with weights (see right side of the figure) and placed on a mattress, with or without a backboard beneath its back (represented by a dark gray rectangle). One triaxial accelerometer was placed on the chest of the manikin and the other beneath its back.

We used two triaxial accelerometers (ADXL330, Analog Devices, Norwood, MA, USA) each one encased in a metal box. One accelerometer was placed on the center of the manikin's chest and the other one beneath its back (Figure 1). During the experiments, we recorded the chest displacement and the two acceleration signals using an acquisition card (NI USB-6211, National Instruments, Austin, TX, USA) connected to a laptop computer, with a sampling rate of 250 Hz and 16-bit resolution.

For this study, we collected a database consisting of forty-eight 3-minute episodes, twelve per couple according to the protocol described in Section 2.1.

### 2.3. Spectral Method for Feedback on Rate and Depth

To estimate the chest and back displacement from the corresponding recorded acceleration values, we applied an algorithm based on the spectral analysis of the acceleration during chest compressions [14]. We designed this algorithm as an alternative to the classical approach of discrete double integration to calculate displacement from acceleration, which presents several drawbacks already discussed in the literature [15, 16]. The algorithm is based on the quasi-periodicity of acceleration during short intervals of chest compressions. Thus, both the acceleration and the displacement can be represented by the first *N* harmonics of their Fourier series decomposition. With this mathematical model, the algorithm provides the mean compression depth and rate achieved by the rescuer every 2 seconds.

**Figure 3:**
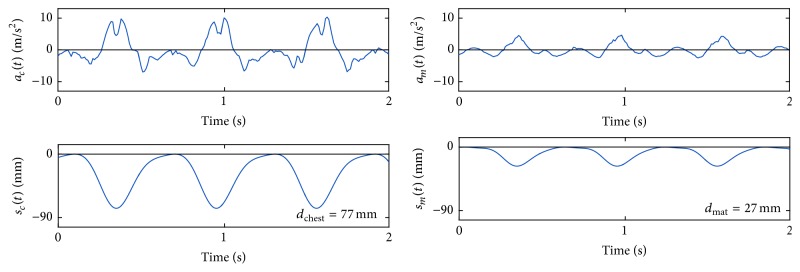
Example of the computation of chest compression depth and rate. The spectral analysis of the chest and back acceleration every 2 seconds allows computing chest displacement and mattress displacement. Subtraction of both values gives the actual chest compression depth, *d*_cc_ = 50 mm. Estimated average rate *r*_cc_ was 99.4 cpm.

Figure 3 shows an example of the method. For each interval, we applied the spectral method to the chest acceleration to compute chest displacement, *d*_chest_, and to the back acceleration to obtain mattress displacement, *d*_mat_. Then, the actual chest compression depth (sternal-spinal displacement) was calculated as the difference between both values: *d*_cc_ = *d*_chest_ − *d*_mat_. Chest compression rate *r*_cc_ corresponded to the fundamental frequency of the chest acceleration.

### 2.4. Data Analysis and Performance Evaluation

Episodes were divided into 2-second consecutive nonoverlapped analysis intervals. The spectral method was applied to every interval to compute one value of depth and rate per interval. These values were compared to the ones obtained after processing the reference compression depth signal (*d*_ref_ and *r*_ref_, resp.). We defined error1acc as the difference between *d*_chest_ and *d*_ref_, that is, the error resulting from using a single chest accelerometer to estimate compression depth. Similarly, we defined error2acc as the difference between *d*_cc_ and *d*_ref_, that is, the error resulting from using two accelerometers to estimate compression depth.

The distributions of the chest and mattress displacement and of the errors in the measurements with one and two accelerometers did not follow a normal distribution according to the Lilliefors test for normality. Values are described by the median and interquartile range (IQR). Wilcoxon rank sum test was used for comparison between two groups, and Kruskal-Wallis test was used for multigroup comparisons. Bonferroni correction was applied to account for multiple comparisons.

## 3. Results

Baseline characteristics of the eight participants selected for the study were women 62% and mean (SD) age 22.5 (1.4) years.  Table 1 shows the median (IQR) computed chest and mattress displacement, the reference chest compression depth, and the unsigned error using one or two accelerometers for the different mattress/backboard combinations. Mattress compression was significantly higher for the sprung surface (*p* < 0.001), and it significantly reduced with the backboard for both surfaces: from 10.4 mm (9.5, 11.4) to 7.0 mm (6.4, 7.4) with *p* < 0.001 and from 37.2 mm (35.0, 40.0) to 24.0 mm (21.6, 27.9) with *p* = 0.002, for foam and sprung mattress, respectively. Global median mattress compression was 17 mm (8, 32).

**Figure 4:**
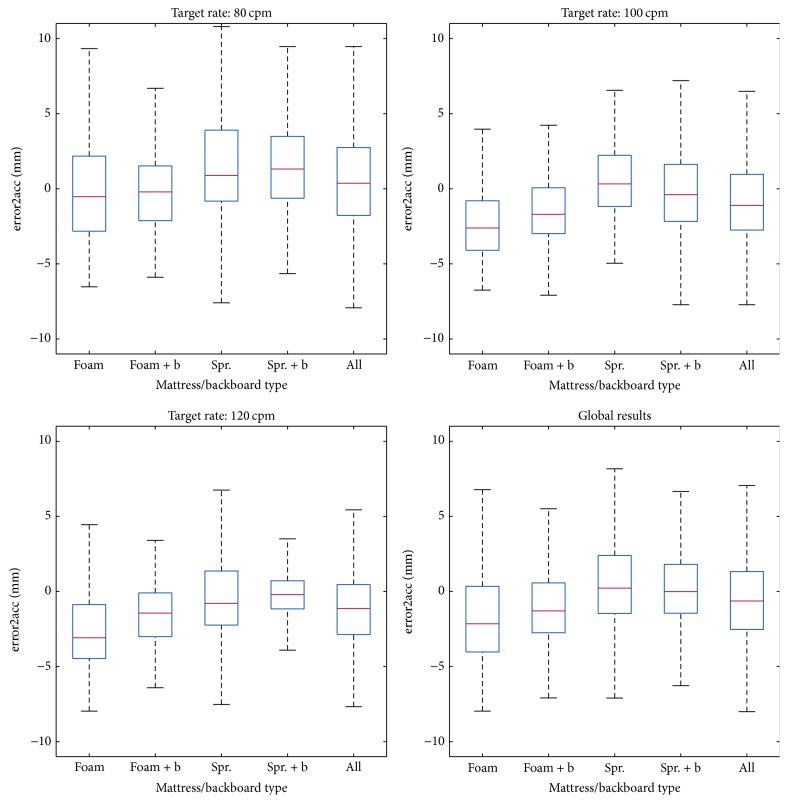
Distribution of the error in depth depending on the mattress/backboard combination. Tested mattresses were foam and sprung (Spr.) The use of a backboard is indicated with + b in the boxplot.

When only the chest acceleration was used, the global median of the error in the estimation of compression depth (error1acc) was 18.1 mm (7.2, 32.8), which corresponded to a percent error of 41.1% (15.3, 72.9). With two accelerometers, the error decreased to 2.1 mm (0.9, 3.6), which corresponded to a percent error of 4.4% (2.0, 7.5). For the different surfaces, median error1acc was 7.2 mm (4.4, 9.9) in the foam and 32.8 mm (25.2, 37.9) in the sprung mattress (*p* < 0.001). Median error2acc decreased to 2.4 mm (1.2, 3.9) in the foam and to 1.7 mm (0.8, 3.2) in the sprung mattress (*p* < 0.001). The use of a backboard significantly affected the results for both surfaces, *p* < 0.001 (see Table 1), but compression rate did not have any significant influence.  Figure 4 shows the distribution of error2acc, as a function of the different mattress/backboard combinations. Results are provided separately for every target rate and globally.

**Figure 5:**
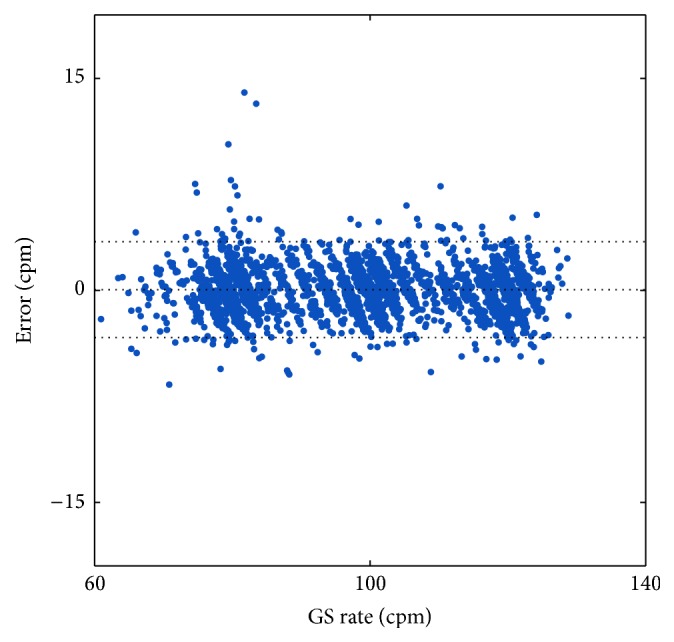
Global distribution of the error in rate estimation. In the modified Bland-Altman plot, dashed lines represent the median of the errors (0.0 cpm) and the 95 percent limits of agreement (−3.3, 3.4 cpm).

Global error in rate estimation was 0.9 cpm (0.4, 1.6), which corresponded to a percent error of 1.0% (0.4, 1.7). No statistically significant differences were found for the different test conditions.  Figure 5 shows the global distribution of the error in rate. In the modified Bland-Altman plot, dashed lines represent the median of the error (0.0 cpm) and the 95 percent limits of agreement (−3.3, 3.4 cpm).

## 4. Discussion

In this study, we proposed a technical solution to provide accurate feedback on chest compression depth and rate when CPR is provided on soft surfaces. The system uses two accelerometers, one placed on the chest and the other beneath the back of the patient. Our algorithm accurately estimated compression depth and rate by spectral analysis of chest and back acceleration proving that CPR feedback on soft surfaces could be feasible.

Global median error of the method with two accelerometers was 2.1 mm (4.4%) in depth and 0.9 cpm (1%) in rate estimation. This performance is comparable to the one reported by the same method when CPR was provided on the floor, with errors below 2 mm and 1.5 cpm [14]. Accurate estimation of compression rate was expected, as it was directly computed as the fundamental frequency of the chest acceleration. In contrast, depth estimation is usually very challenging in this scenario. The accuracy of our method in the different test conditions, however, proved its stability (median error below 3.2 in all cases).

Our results confirmed the compression depth overestimation of single-accelerometer-based CPR devices when used on soft surfaces. Global median chest displacement, that is, estimated compression depth with a single accelerometer, was 62 mm, whereas the actual compression depth was 47 mm (Table 1). This led to an unacceptable median error of 41%. Other studies reached similar results: Beesems and Koster evaluated the performance of a commercial accelerometer-based CPR device [17], reporting a measured depth of 54 mm (foam) and 56 mm (air mattress), compared to the manikin's reference of 42 mm and 35 mm, respectively.

Using one accelerometer, results were different depending on the type of mattress. Delivering compressions with the manikin on the sprung mattress was much more difficult than on the foam one. The sprung mattress presented less stiffness, and consequently mattress compression was much higher than with the foam one. In addition, participants had difficulties generating the required downward chest displacement (perpendicular to the chest) with the sprung mattress. Acceleration was observed in the *x*-axis and *y*-axis of the chest sensor not contributing to the vertical movement. This could explain the very high overestimation of chest compression depth with a single accelerometer (median error1acc 32.8 mm), even with the backboard. With two accelerometers, however, mattress displacement was very accurately compensated in both surfaces. Error (error2acc) decreased drastically to 2.4 mm in the foam and 1.7 mm in the sprung. Mattress sinking and lateral movements were very well compensated between the two sensors and thus accuracy increased.

Aase and Myklebust suggested in 2002 [16] the use of two accelerometers to estimate chest compression depth in moving environments. One accelerometer measured chest acceleration and the other one floor acceleration. In that study, both recorded acceleration values were subtracted before applying the algorithm for computing chest compression depth. Oh et al. [18] applied integration and detrending to chest and back acceleration for computing chest and mattress displacement waveforms, respectively. In both approaches, the difficulty lies in that when two oscillating signals (acceleration or displacement) are added or subtracted, an error in the synchronization of the signals would introduce a phase error and could significantly modify the waveform of the resulting signal. Small asynchrony between both signals would result in unacceptable errors in the estimation of compression depth. For tight synchronization, these approaches would require a wired connection between the two accelerometers which could complicate the practical implementation.

In contrast, our algorithm processes independently consecutive intervals of each acceleration. Chest and mattress displacement values are separately computed and then subtracted every 2 seconds. This approach is simpler and eliminates the need for fine synchronization between the two sensors. Even if the analysis time intervals were not perfectly aligned, the error in the subtraction of both computed distances would be small. This could allow wireless communication between both accelerometers.

This study presented some limitations. First, we used a single resuscitation manikin. Even though we increased its weight to provide a more realistic simulation of a human torso, manikin differs in stiffness from a human chest, and there are also wide variations in the morphology of human chest. However, our simulated experimental setup provided a framework to test the method's accuracy in a wide range of test conditions. Second, the accuracy of the method could vary if CPR were provided by experts, especially if they are familiarized with in-hospital compressions. A well consolidated CPR technique with a more stable acceleration pattern could increase the algorithm's accuracy. The clinical applicability of our proposal would therefore require prospective validation studies with CPR experts, more surfaces, and different patients.

## 5. Conclusion

The system described in this study provided accurate feedback on chest compression depth and rate during CPR administered on two types of mattresses, foam and sprung, using two accelerometers and spectral analysis of the acceleration. Error in the estimation of compression depth was significantly reduced with respect to that reported with a single accelerometer. Our solution compensated mattress displacement, avoiding overestimation of compression depth when CPR is performed on soft surfaces. Quality of chest compressions in these scenarios could therefore be enhanced to adhere to resuscitation guidelines recommendation.

## Figures and Tables

**Table 1:** Computed chest and mattress displacement (*d*_chest_ and *d*_mat_), reference chest compression depth (*d*_ref_), and unsigned error in the estimation of the spinal-sternal displacement with one accelerometer (error1acc) and two accelerometers (error2acc), for different mattress/backboard combinations.

Mattress	Parameter (mm)				
	*d*_chest_	*d*_mat_	*d*_ref_	error1acc	*error2acc*
*Foam*					
Backboard	55.6 (51.7, 58.8)	7.0 (6.4, 7.4)	48.5 (45.4, 51.6)	4.5 (2.8, 6.8)	*3.1 (1.5, 4.5)*
No backboard	56.6 (52.1, 60.2)	10.4 (9.5, 11.4)	46.0 (42.0, 48.9)	9.1 (7.4, 11.2)	*2.0 (0.9, 3.1)*
*Sprung*					
Backboard	69.9 (62.3, 79.6)	24.0 (21.6, 27.9)	45.8 (40.5, 51.8)	25.2 (22.4, 29.0)	*1.9 (0.9, 3.3)*
No backboard	83.4 (78.4, 89.1)	37.2 (35.0, 40.0)	46.0 (42.6, 49.7)	37.7 (34.9, 41.4)	*1.6 (0.6, 3.1)*
*Global*	61.9 (55.5, 78.7)	17.0 (8.0, 32.5)	46.7 (42.7, 50.4)	18.1 (7.2, 32.8)	*2.1 (0.9, 3.6)*

## References

[1] Abella B. S., Alvarado J. P., Myklebust H. (2005). Quality of cardiopulmonary resuscitation during in-hospital cardiac arrest. *The Journal of the American Medical Association*.

[2] Perkins G. D., Benny R., Giles S., Gao F., Tweed M. J. (2003). Do different mattresses affect the quality of cardiopulmonary resuscitation?. *Intensive Care Medicine*.

[3] Noordergraaf G. J., Paulussen I. W. F., Venema A. (2009). The impact of compliant surfaces on in-hospital chest compressions: effects of common mattresses and a backboard. *Resuscitation*.

[4] Perkins G. D., Handley A. J., Koster R. W. (2015). European Resuscitation Council Guidelines for Resuscitation 2015: section 2. Adult basic life support and automated external defibrillation. *Resuscitation*.

[5] Travers A. H., Perkins G. D., Berg R. A. (2015). Part 3: adult basic life support and automated external defibrillation: 2015 international consensus on cardiopulmonary resuscitation and emergency cardiovascular care science with treatment recommendations. *Circulation*.

[6] Andersen L. Ø., Isbye D. L., Rasmussen L. S. (2007). Increasing compression depth during manikin CPR using a simple backboard. *Acta Anaesthesiologica Scandinavica*.

[7] Fischer E. J., Mayrand K., Ten Eyck R. P. (2016). Effect of a backboard on compression depth during cardiac arrest in the ED: a simulation study. *The American Journal of Emergency Medicine*.

[8] Perkins G. D., Smith C. M., Augre C. (2006). Effects of a backboard, bed height, and operator position on compression depth during simulated resuscitation. *Intensive Care Medicine*.

[9] Putzer G., Fiala A., Braun P. (2016). Manual versus mechanical chest compressions on surfaces of varying softness with or without backboards: a randomized, crossover manikin study. *The Journal of Emergency Medicine*.

[10] Meaney P. A., Bobrow B. J., Mancini M. E. (2013). Cardiopulmonary resuscitation quality: improving cardiac resuscitation outcomes both inside and outside the hospital: a consensus statement from the American heart association. *Circulation*.

[11] Gruber J., Stumpf D., Zapletal B., Neuhold S., Fischer H. (2012). Real-time feedback systems in CPR. *Trends in Anaesthesia and Critical Care*.

[12] Lee S., Oh J., Kang H. (2015). Proper target depth of an accelerometer-based feedback device during CPR performed on a hospital bed: a randomized simulation study. *The American Journal of Emergency Medicine*.

[13] Nishisaki A., Nysaether J., Sutton R. (2009). Effect of mattress deflection on CPR quality assessment for older children and adolescents. *Resuscitation*.

[14] González-Otero D. M., Ruiz J., Ruiz De Gauna S., Irusta U., Ayala U., Alonso E. (2014). A new method for feedback on the quality of chest compressions during cardiopulmonary resuscitation. *BioMed Research International*.

[15] Ruiz de Gauna S., González-Otero D. M., Ruiz J., Russell J. K., Groeneveld A. J. (2016). Feedback on the rate and depth of chest compressions during cardiopulmonary resuscitation using only accelerometers. *PLoS ONE*.

[16] Aase S. O., Myklebust H. (2002). Compression depth estimation for CPR quality assessment using DSP on accelerometer signals. *IEEE Transactions on Biomedical Engineering*.

[17] Beesems S. G., Koster R. W. (2014). Accurate feedback of chest compression depth on a manikin on a soft surface with correction for total body displacement. *Resuscitation*.

[18] Oh J., Song Y., Kang B. (2012). The use of dual accelerometers improves measurement of chest compression depth. *Resuscitation*.

